# Naming and Knowing Revisited: Eyetracking Correlates of Anomia in Progressive Aphasia

**DOI:** 10.3389/fnhum.2019.00354

**Published:** 2019-10-11

**Authors:** Molly B. Ungrady, Maurice Flurie, Bonnie M. Zuckerman, Daniel Mirman, Jamie Reilly

**Affiliations:** ^1^Penn Frontotemporal Degeneration Center, University of Pennsylvania, Philadelphia, PA, United States; ^2^Eleanor M. Saffran Center for Cognitive Neuroscience, Temple University, Philadelphia, PA, United States; ^3^Department of Communication Sciences and Disorders, Temple University, Philadelphia, PA, United States; ^4^Department of Psychology, The University of Edinburgh, Edinburgh, United Kingdom

**Keywords:** dementia, primary progressive aphasia, language treatment, language disorder, anomia, eye tracking

## Abstract

Progressive naming impairment (i.e., anomia) is a core diagnostic symptom of numerous pathologies that impact anterior and inferior portions of the temporal lobe. For patients who experience such regional temporal lobe degeneration, patterns of language loss often parallel the degradation of semantic memory, an etiology of naming impairment known as semantic anomia. Previous studies of semantic anomia have focused extensively on the output of naming attempts by contrasting errors, omissions, and distortions as a function of item-level characteristics (e.g., prototypicality, semantic category). An alternative approach involves evaluating visual confrontation naming as the naming process unfolds. Techniques with high temporal resolution (e.g., eyetracking) offer a potentially sensitive mode of delineating the locus of impairment during naming. For example, a lexical retrieval disorder would hypothetically elicit normal gaze patterns associated with successful visual object recognition regardless of naming accuracy. In contrast, we hypothesize that semantic anomia would be distinguished by aberrant gaze patterns as a function of reduced top-down conceptually guided search. Here we examined visual object recognition during picture confrontation naming by contrasting gaze patterns time locked to stimulus onset. Patients included a cohort of patients with anomia associated with either primary progressive aphasia (*N* = 9) or Alzheimer’s disease (*N* = 1) who attempted to name 200 pictures over the course of 18–24 months. We retrospectively isolated correct and incorrect naming attempts and contrasted gaze patterns for accurate vs. inaccurate attempts to discern whether gaze patterns are predictive of language forgetting. Patients tended to show a lower fixation count, higher saccade count, and slower saccade velocity for items that were named incorrectly. These results hold promise for the utility of eyetracking as a diagnostic and therapeutic index of language functioning.

## Introduction

Neurotypical adults can name objects and people with high accuracy and little cognitive effort. However, the ease by which naming unfolds belies the complexity of the cognitive process. Successful confrontation naming (i.e., producing a target name when presented with a picture) demands the precise orchestration of a chain of interactive processes, beginning with visual object recognition, proceeding through semantic processing and lexical retrieval, ultimately resulting in overt articulation. Anomia, or the inability to name common objects and people, can result from disturbances at any stage of this process and is often one of the most functionally debilitating symptoms of living with a neurodegenerative disorder (Laine and Martin, 2006; Henry et al., 2008; Flanagan et al., 2016; Reilly, 2016).

The etiology of impairment in anomia often, but not always, manifests as a distinctive error pattern. The study of naming errors and the treatment of naming disorders in acquired neurogenic language disorders has historically fallen within the purview of aphasiology. Anomia in classical cortical aphasia syndromes is thought to reflect impaired linguistic access to otherwise intact conceptual knowledge (Warrington and Shallice, 1979, 1984; Shallice et al., 1983; Mirman and Britt, 2014). Anomia in post-stroke aphasia tends to manifest in a relatively inconsistent manner on a trial-by-trial basis (e.g., “dog” may be erroneously named as “doll” on one trial and successfully named on another trial). In contrast, anomia in dementia with progressive semantic impairment generally tends to have different properties in terms of its etiology, response consistency, and progression. Hereafter, we refer to this particular etiology of impairment as *semantic anomia*. Semantic anomia does not include a lexical access impairment, but rather is characterized by a loss of object knowledge. Hurley et al. (2012) also found these two distinct patterns of impairments in groups of PPA: lexical access and lexical semantic.

Although this etiology is common in disorders with progressive semantic impairment, it is also not unusual to see a combination of both semantic and lexical access impairments (Mesulam et al., 2009). In its mild stages, Mesulam et al. (2009) find that the semantic variant of Primary Progressive Aphasia (svPPA) starts out as a lexical access impairment, as the patients are able to recognize words but unable to produce the words. As the disease progresses, the patients lose their ability to even recognize the objects, and the semantic anomia becomes a prominent symptom. Thus, once the initial stages of the disease have passed, the inability to name an object likely indicates a semantic breakdown, even if lexical access impairments might be present as well.

One characteristic of semantic anomia, according to Hodges et al. (1996), is a dichotomy between “naming vs. knowing.” In this work, the authors examined the quality of concept definitions for items that patients with Alzheimer’s disease (AD) could successfully name relative to their anomic target items. The principal finding was that of globally impoverished concept definitions for items that could not be named, a pattern that established a strong correlation between residual semantic knowledge and naming accuracy.

In a related task, Bozeat et al. (2003) examined the correlation between naming and knowing in a longitudinal study of object drawing in svPPA. Patients produced unique errors in the production of line drawings of concrete concepts (e.g., duck, lamp) to a verbally cued label. Comparisons of line drawings produced over time demonstrated a progressive loss of distinctive semantic features, consistent with feature dimming or averaging to the central tendency of a prototype. For example, as semantic impairment worsened, patients added two additional legs to the drawing of a duck, approximating its form as a prototypical four-legged animal. Patients showed a significant association between drawing performance, object naming, and word-to-picture matching. These results provide converging evidence that “knowing” deficits that occur in the presence of progressive semantic impairments compromise a range of verbal and non-verbal abilities, further differentiating progressive aphasia from stroke aphasia.

A reliably strong correlation between naming and knowing confers significant inference to naming ability. If indeed anomia observed in progressive aphasia is predominantly characterized by semantic impairment, then naming ability in this population can reasonably provide a proxy measure for the integrity of semantic knowledge (Reilly et al., 2011a, b). In contrast, inconsistency of naming errors in stroke aphasia and the nature of stroke as an access impairment preclude or at the very least jeopardize the reliability of such inference (Malt, 2019).

Much of the inference gleaned of semantic memory from the analysis of naming is derived from studies of output. Analyses of naming errors lend complementary detail about the processes and mechanisms underlying such output impairments. For example, a disproportionate impairment in the ability to name biological natural kinds relative to manufactured artifacts is one of the hallmarks of category-specific naming impairments associated with AD (Farah and McClelland, 1991; Caramazza and Shelton, 1998; Laws and Sartori, 2005; Lambon Ralph et al., 2007). Furthermore, referring to a knife as “you cut with it” or as a “kitchen thing” demonstrates preserved functional and thematic knowledge in the context of inaccurate retrieval. Despite the inferential value of error and item analyses, an exclusive focus on output affords limited inference about the mechanism underlying anomia. Consider, for example, a retrospective attempt to reconstruct the complex series of events leading up to a ruined recipe. You observe a ruined cake, but at what stage did the process fail? Often, the only possible way to answer to this question requires a perspective that evaluates success or failure of each step in real time as they are added to the recipe. In the domain of visual confrontation naming, eyetracking offers a powerful means of forward inference.

In past studies, eyetracking has provided insights into normal processes that underlie naming (e.g., picture identification, semantic categorization). The visual world paradigm (VWP), for example, involves analyzing gaze patterns to a particular scene or sequence of photographs while hearing verbal descriptors (Cooper, 1974; Tanenhaus et al., 1995). Among the first to study the VWP was Cooper, who found that as we hear a phrase such as “my scatterbrained dog Scotty…” gaze tends to focus on a picture of a dog more than on other unrelated pictures. When they subsequently hear a phrase about a “photographic safari,” their gaze moves from the dog toward the picture of a camera. These findings confirmed that eyetracking tracks eye movement time-locked to verbal cues, and thus provides a unique, time sensitive window into cognitive processes. Similar to this connection between words we hear and gaze patterns, research shows strong connections between words we speak and gaze patterns, with fixation to an object occurring less than 1000 ms before the verbal description of an object (Meyer et al., 1998; Griffin and Bock, 2000). This informs us about the process of naming: first visualize the object, then comprehend the object, next choose the word from the mental lexicon, and finally produce its phonological form. It also demonstrates that the amount of time that a speaker spends fixating on a particular object is contingent on how long they need to complete this process (Griffin, 2001). For example, Meyer et al. (2003) demonstrated that neurotypical adults exhibited a longer fixation duration at an object with a longer and more difficult name than at an object with a shorter and easier name. These studies, among others, support that eyetracking is a tool that allows us insight into the complex process of naming.

In addition to normal or neurotypical processing, eyetracking has also proven useful as a metric for identifying and distinguishing between neurological disorders. For example, posterior cortical atrophy (PCA), also known as the visual variant of AD, is characterized by atypical plaque density within the primary visual cortex (Crutch et al., 2012). PCA patients tend to experience some degree of apperceptive and/or associative visual agnosia (Benson et al., 1988). Shakespeare et al. (2015) identified unique saccade behaviors in PCA patients when compared to typical AD and control subjects in a series of eyetracking assessments of stationary and moving fixation tasks. Specifically, PCA saccadic behaviors were slower and less efficient compared to typical amnestic AD patients. In regard to impairment in AD overall, both PCA and typical AD groups were characterized by reduced fixation stability (i.e., eccentricity in gaze around a focal point). When coupling eyetracking behaviors with volumetric MRI scans, authors were able to suggest different foundational causes for such aberrant fixation responses in each group. Reduced fixation stability in PCA patients was associated with a high frequency of large saccadic intrusions and reduced cortical thickness – suggesting a cognitive foundation for fixation impairment rooted in higher cortical processing. This work not only demonstrates the potential for eyetracking as a tool for identifying impairments but also as a sensitive measure of AD subtypes and their underlying relationships to cognitive processes.

Regarding the semantic anomic patients, studies have shown that eye gaze patterns reveal information about the underlying mechanisms of naming, and thus provide insight into the process of semantic representation. Rösler et al. (2000) demonstrated that in a visual search task where AD patients were asked to find a number or letter target amongst 79 number or letter distractors, the AD patients exhibited a higher number of fixations, longer fixation durations, and a delayed response time compared to age-matched neurotypical controls. This suggests an inefficient visual search strategy in semantic anomic AD cases (Rösler et al., 2000), as they were unable to efficiently plan a search with minimal fixations organized by a top-down visual search strategy. In PPA, Seckin et al. (2016) recently evaluated the VWP as a means of exploring information processing in a word-to-object matching task. Individuals with PPA were asked to select an object from a circular array that matched a previously presented word (i.e., individuals observed a target word and were instructed to select the matching object image from a selection of 16 object probes). Results indicated that the PPA group demonstrated increased “back and forth” gazing behavior between related foils when compared to controls – offering evidence for bottom-up “probabilistic” mapping in selection rather than an efficient, decisive mapping between semantically matched object probes and word targets.

Previous investigations of naming and knowing have been guided by an offline, output-based empirical perspective such as analyzing correlations between naming with concept definitions, drawing, and word-to-picture matching. Here we employed eyetracking during confrontation naming as a means for affording forward inference about the locus of impairment in semantic anomia. We work from the assumption that anomia in the PPA and AD cases in the current study has a primary etiology of semantic loss, based on previous literature (Caramazza and Shelton, 1998; Reilly et al., 2011a). As such, we hypothesize that patients “know” less about words they cannot accurately name. This dearth of knowledge will impact patterns of visual search during picture naming such that patients will struggle to rapidly fixate on key diagnostic features of items they do not know and cannot name. Therefore, we predict that unnamed items will be subjected to an inefficient search path comprised of more fixations (e.g., looking at many irrelevant features), increased number of saccades (e.g., more undirected looking around), and slower saccade velocity (e.g., unguided search and thus slower to reach a feature).

## Materials and Methods

### Overview

We tracked eye movements as participants with progressive naming impairment associated with either AD (*N* = 1) or PPA (*N* = 9) named common objects and familiar and famous people. In the first analysis we used a logistic mixed effects approach to isolate and contrast eye gaze patterns for words with accurate vs. inaccurate responses. In a second analysis we correlated neuropsychological measures of language and memory from the same time points with eyetracking and naming accuracy measures.

### Patients

We included patients with the primary amnestic variant of AD and PPA. Patients included nine patients with PPA and one patient with AD tested over the span of 18–24 months. Demographic and neuropsychological data appear in Table 1.

**TABLE 1:** Patient demographics and neuropsychological tests.

**Patient**	**Dx**	**Age**	**Year Onset**	**Edu**	**BNT**	**PPTpics**	**PPTwords**	**MoCA**	**DigitsF**	**DigitsB**	**TrailATime**	**TrailBTime**
S01	svPPA	60–65	2011	15	1.4 (0.55)	17.2 (7.33)	13.25 (2.99)	9.6 (3.85)	5.8 (0.84)	5.2 (0.45)	105.68 (37.45)	281.6 (47.69)
S02	lvPPA	66–70	2012	13	10.33 (2.09)	25.34 (0.58)	25.34 (0.58)	15.5 (0.71)	6 (1.42)	5 (1.42)	58 (12.73)	136.5 (26.17)
S03	lvPPA	60–65	2011	14	12.2 (1.49)	22.6 (1.68)	23.8 (0.45)	12.8 (2.29)	3.25 (0.5)	2 (0)	59.86 (24.87)	289.5 (21)
S04	AD	76–80	2000	16	4.25 (0.5)	19.75 (3.41)	18.25 (2.07)	13.5 (3.11)	8 (1.42)	6.75 (0.5)	30.29 (6.77)	70.22 (20.06)
S05	svPPA	60–65	2012	18	3.2 (0.84)	21 (1.59)	22.8 (1.93)	19.4 (1.95)	6.8 (0.84)	7.4 (1.15)	32.64 (6.63)	89.24 (9.52)
S06	svPPA	60–65	2009	19	1.75 (0.5)	13.75 (1.5)	13.75 (1.71)	16.5 (1.3)	10.75 (0.96)	9 (0.82)	49.83 (5.8)	102.73 (10.14)
S07	svPPA	60–65	2013	12	4 (0)	22 (2.17)	18.8 (2.39)	16.6 (2.51)	5 (1.23)	6 (0.71)	19.62 (2.92)	49.74 (9.78)
S08	svPPA	66–70	2015	16	3.75 (0.5)	21.25 (2.07)	18.34 (1.16)	18.75 (2.22)	6.25 (0.5)	7.5 (1.74)	32.11 (8.83)	50.99 (6.59)
S09	svPPA	56–60	2011	12	1.5 (0.58)	23.25 (1.5)	17 (4.25)	19.25 (1.5)	8.67 (0.58)	8.34 (1.53)	26.47 (11.25)	63.88 (14.32)
S10	svPPA	60–65	2010	16	3.5 (0.71)	21.67 (0.58)	20.5 (2.13)	17.5 (2.13)	6 (1.42)	5.5 (0.71)	40.19 (0.15)	113.24 (6.7)
Max/Norm	NA	NA	NA	NA	15/13.2	26	26	30/26	9	8	NA	NA

*Neuropsychological performance, M(SD) of patients across all time points. Dx, Diagnosis (svPPA, semantic variant Primary Progressive Aphasia; lvPPA, logopenic variant Primary Progressive Aphasia; AD, Alzheimer’s Disease), Age, Age at baseline; YearOnset, Year diagnosed; Edu, Years of Education, BNT, Boston Naming Test Score; PPTPics, Pyramids and Palm Trees Test Score-Pictures; PPTwords, Pyramids and Palm Trees Test Score-Words; MoCA, Montreal Cognitive Assessment Score; DigitsF, Digit Span-Forward Score; DigitsB, Digit Span-Backward Score; TrailATime, Trail Making Test-A time to complete (seconds), TrailBTime, Trial Making Test-B time to complete (seconds). In the last row, we included the maximum score for each test and gave means based on normative data where applicable (Mack et al., 1992; Nasreddine et al., 2005).*

Among the PPA patients, diagnoses were first established by experienced behavioral neurologists and later confirmed using a consensus approach based on the Gorno-Tempini et al. (2011) diagnostic criteria. The cohort included seven patients with svPPA and two patients with logopenic variant (lvPPA). One patient had a diagnosis of AD established using the McKhann et al. (2011) criteria. Each participant was enrolled for 18–24 months, completing baseline testing upon enrollment and then follow-up testing every 6 months. At the testing sessions, patients completed a battery of neuropsychological tasks, which included Digits Forward and Backward (Wechsler, 2009), Trails A and B (War Department Adjutant General’s Office, 1944), the Montreal Cognitive Assessment (MoCA) (Nasreddine et al., 2005), the brief (15-item) form of the Boston Naming Test (BNT) (Kaplan et al., 1983; Mack et al., 1992), and Pyramids and Palm Trees (Howard and Patterson, 1992). We assessed naming at each time point for a combination of control (assigned randomly) and personalized (personal items and family members) picture stimuli (*N* = 200).

### Eyetracking Procedures

We tracked eye movements using an infrared, laptop-mounted eyetracking system (SMI iView X RED eye-tracker) (SensoMotoric Instruments Inc., Boston, MA, United States). We presented picture stimuli using SMI’s proprietary software (Experiment Center) and tracked movements of the right eye at a sampling rate of 120 Hz (spatial resolution < 0.03°). Patients were seated at a distance of 55–65 cm away from the infrared illuminator bar positioned at the bottom of the laptop monitor. Each eyetracking session initiated with a 5-point calibration and validation procedure. The SMI RED eyetracker uses a low-speed event detection algorithm to define fixations and saccades. This method considers fixations as its primary event and derives information about saccades based on the fixations. This algorithm considers a group of consecutive points within a particular dispersion, over a defined amount of time as a fixation. We used the default parameters for this definition, with a fixation event defined as when the consecutive points have a maximum dispersion of 100px and a minimum duration of 80 ms.

### Picture Stimuli

Each participant selected a personal lexicon of 100 words that were used with high frequency in their daily lives, broken down into seven categories: people (e.g., their spouse), places (e.g., their church), foods (e.g., bananas), household items (e.g., television), hygiene items (e.g., toothbrush), clothes (e.g., shorts), and activities (e.g., exercising) (for item selection criteria see Reilly, 2016). Once these words were chosen by the participant and their families, pictures of these items were taken in their homes of their own personal items, henceforth referred to as “trained images.” They were then edited, adapted to a laptop, randomized, and presented individually, while the eye tracker recorded their gaze patterns. The onset of each picture was prompted via gaze contingency, where the eye tracker accrued gaze for 1000 ms within a rectangular area of interest (AOI) at the top of the screen prior to the onset of the next stimulus. In addition, each participant was assigned a set of 100 untrained images that served as a control condition. Trained and untrained stimuli were presented in separate blocks using the same stimulus presentation parameters as the trained images. Patients were assigned trained and untrained items using the procedures outlined by Reilly (2016). That is, patients together with their caregivers reviewed fixed lists of words blocked by semantic category (e.g., clothes, hygiene items). Approximately half of the words were chosen by the patient and their caregivers as training targets, whereas the remainder served as untrained controls. Thus, each patient had a different set of trained and untrained items depending personal preference. Both conditions, trained and control images, were included in analysis and collapsed across conditions.

### Naming Procedures and Scoring

Patients were asked to verbally state the name of the items in all of the pictures after the presentation of the image. They were allowed unlimited time to provide an answer. When necessary patients were cued semantically first, phonologically second, however, only the spontaneous response was scored as either accurate or inaccurate. If the participant self-corrected their spontaneous response, we considered their self-correction as the response to score. We utilized a binary scoring protocol, where responses were either correct or incorrect. Patients were asked to name both sets of pictures, the personalized lexicon and the canonical images, at baseline, and then every 6 (±2) months for up to 2 years.

### Eyetracking Metrics

All eyetracking data were windowed to 2750 ± 250 ms upon presentation of the stimulus in order to control for differences in patterns that could result from analyzing a wide range of presentation time (i.e., a stimulus that was viewed for 200 ms vs. a stimulus that was viewed for 5000 ms). When extracting the data from SMI BeGaze, we exported the eyetracking data from the first 3000 ms, and then further restricted the presentation time in RStudio to 2750 ± 250 ms prior to analysis.

Eyetracking studies of scene viewing tend to encompass measures of depth and breadth of visual attention (e.g., where is someone looking). Depth of visual attention is typically indexed by fixation measures (e.g., count, duration) which are thought to quantify deep processing of particular elements of scenes (e.g., faces). In contrast, breadth of search is indexed by saccade measures (e.g., count, amplitude). Since we are interested both in the depth and breadth of visual search, we analyzed a range of fixation and saccade measures commonly used in scene perception research. These included fixation count, fixation duration total, fixation dispersion total, saccade count, saccade duration total, saccade amplitude total, and saccade velocity total. Upon visual inspection of the data, we isolated four eye gaze metrics. The first measure was fixation count, which includes the total number of fixations that occurred within the windowed timeframe. Second, we assessed the fixation dispersion. However, this measure was highly correlated with fixation count (*r* = 0.95) and due to potential multicollinearity was not included in the analysis. Next we assessed the number of saccades (i.e., saccade count). Finally, we evaluated saccade velocity, defined by the change in eye position (degrees) divided by seconds. For this study, we divided the saccade velocity by 100 in order to keep the unit (ms) between each of our variables consistent.

### Data Analysis and Statistical Procedures

We employed a logistic mixed effects model to assess predictors of item-level accuracy using the “lme4” package within R, collapsing across condition (i.e., trained or untrained pictures) and time points. Fixed effects included: number of fixations, number of saccades, and saccade velocity. Random effects included participant and item. To evaluate the unique contribution of each of the fixation measures to model fit, each measure was iteratively removed from the model while leaving the other two fixed effects in the model. That is, each fixation measure’s unique contribution to model fit was assessed while controlling for the other fixation measures. Changes in goodness of model fit were assessed by the likelihood ratio test: two times the change in log-likelihood, which is distributed as χ^2^ with degrees of freedom corresponding to the difference in number of parameters (one in each of these comparisons).

In order to characterize the association of eyetracking behaviors with various measures of cognition, we performed a correlation analysis between eyetracking performance and neuropsychological performance. Pearson correlations were generated based on mean performance of each participant at each timepoint. Incomplete observations were removed from analysis.

The dataset for this study can be found in Open Science Framework^1^.

## Results

Each of the eyetracking measures were significant predictors of naming accuracy. Table 2 reflects output of the logistic mixed effects model predicting item accuracy.

**TABLE 2:** Mixed logistic regression.

	**Estimate (SE)**	**χ^2^(1)**	***p***
Number of fixations	0.065 (0.018)	12.6	<0.001
Number of saccades	−0.0919 (0.018)	26.0	<0.0001
Saccade velocity	0.0496 (0.0085)	33.3	<0.0001

*This table shows the results of the mixed logistic regression. The number of fixations, the number of saccades, and the saccade velocity were all significant predictors of inaccurate naming. This analysis was completed in R and the syntax for the model was “glmer(Acc ∼ Fixation Count + Saccade Count + Saccade Velocity + (1| Patient) + (1| Item), data = , family = binomial).”*

**FIGURE 1:**
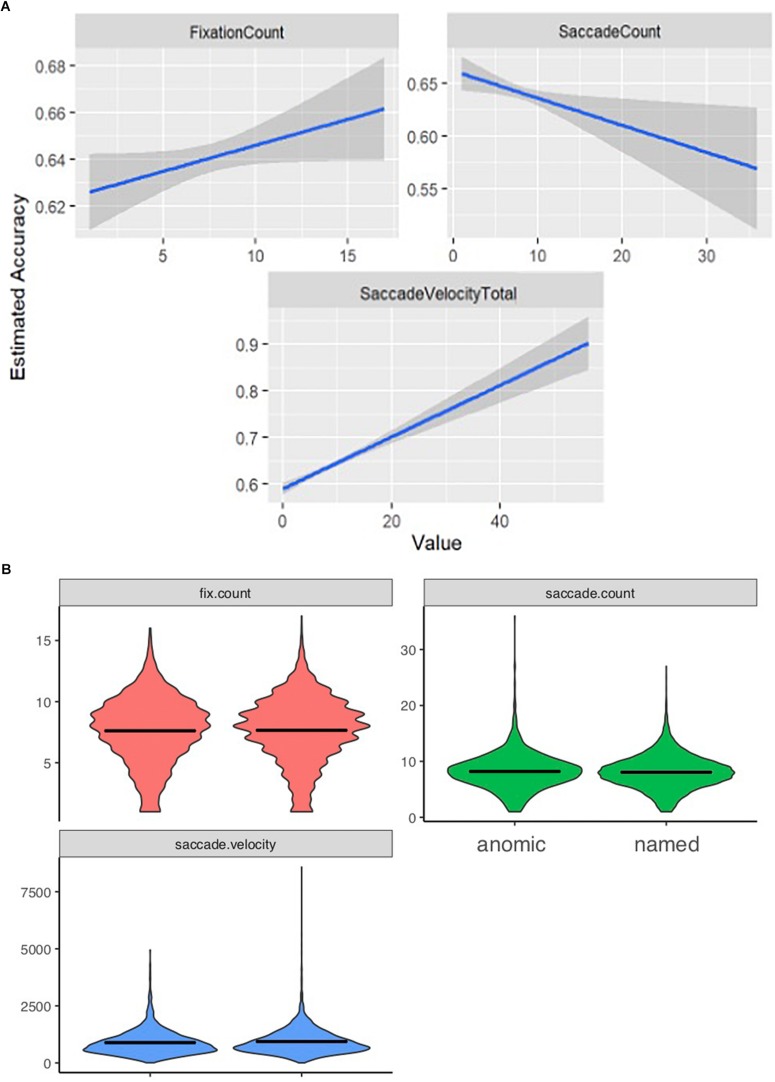
Eyetracking patterns based on accuracy. Panels **(A)** shows how the eyetracking measures predict accuracy. As accuracy is lower patients displayed a lower fixation count; a higher saccade count, and a slower saccade velocity. **(B)** Represents the range and distribution of data observed for each eyetracking measure [fix.count (fixation count), saccade count, and saccade velocity] between the anomic (items inaccurately named) and named (items accurately named) items.

Total number of fixations was significantly lower for incorrectly named items (mean = 7.61, SD = 2.75) than for correctly named items (mean = 7.65, SD = 2.70; *p* < 0.001). The total number of saccades produced during the viewing of incorrectly named images (mean = 8.20, SD = 3.22) was significantly higher than the number of saccades for correct responses (mean = 8.06, SD = 2.97; *p* < 0.001). Finally, saccade velocity for the incorrectly named items (mean = 890.76, SD = 3.22) was significantly slower than for the correctly named items (mean = 937.34, SD = 549.08; *p* < 0.001). Model-predicted associations between each of these measures and accuracy are shown in Figure 1A. To further describe the data, we added a violin plot (Figure 1B) of each eyetracking variable.

We see that a lower fixation count, a slower saccade velocity and a higher saccade count are significant predictors of lower accuracy, and thus impaired knowing.

### Neuropsychological Performance and Naming Inaccuracy

**FIGURE 2:**
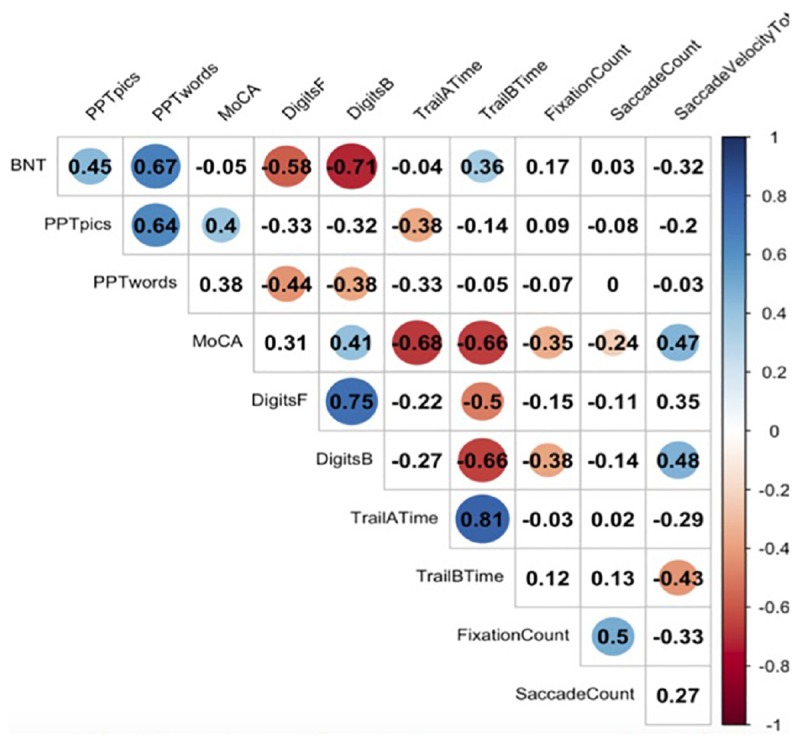
Correlations between eyetracking metrics and neuropsychological tasks. Here we see significant correlations between neuropsychological measures and eyetracking measures. A blue dot indicates a positive correlation, and a red dot indicates a negative correlation. Numbers reflect Pearson correlation coefficients.

Figure 2 represents a correlation matrix describing relationships between gaze metrics and offline neuropsychological measures for incorrectly named items. Global cognitive performance as measured by the MoCA was moderately positively correlated with total number of fixations (*r* = −0.35, *p* = 0.03), total number of saccades (*r* = −0.24, *p* = 0.03), and total saccade velocity (*r* = 0.47, *p* = 0.01), indicating that reductions in global cognition were associated with more diffuse gaze, less focused attention, and slower patterns of looking at points between attentional fixation. Executive functioning as indexed by Digit Span-Backward was positively correlated with saccade velocity total (*r* = 0.48, *p* = 0.03).

We observed no significant correlations between offline measures of naming performance, semantic memory, and gaze.

## Discussion

We examined eyetracking as a sensitive measure for evaluating naming in the context of progressive semantic impairment. Little is known about how semantic impairment impacts visual object recognition and how these associated gaze patterns predict naming accuracy. Our working hypothesis is that visual confrontation naming involves a combination of top-down expectancies and bottom-up, salience driven processing. This hypothesis was also tested by Choi et al. (2017) who found that neurotypical older adults were just as easily able to access top-down and bottom-up strategies as younger adults in order to optimize reading strategy.

A global deficit in semantic processing would, therefore, reduce the top-down contribution, forcing reliance upon bottom-up salience. This division of labor between conceptual expectancy and sensory-driven visual search has been reported in other experimental paradigms (Yarbus, 1967). However, a comprehensive account of gaze behaviors that parallels semantic degradation during naming is lacking.

The following gaze patterns differentiated known (named accurately) from forgotten (anomic) items: saccade count, fixation count, and saccade velocity. Specifically, forgotten items were associated with a lower fixation count, slower saccade velocity, and an increased number of saccades. Scan paths for forgotten items appeared unguided and disorganized with unstable gaze patterns. That is, the eyes are not staying still long enough to constitute a fixation, and instead moving around enough to be counted as saccades.

A few aberrant gaze patterns could likely arise as the result of impoverished semantic knowledge in picture naming. Due to a loss of top-down knowledge of an object, patients experiencing semantic anomia might engage in a bottom-up driven search, thus resulting in disorganized searching around the image to identify the diagnostic and salient features of an object. This would result in increased fixation and saccadic occurrences. Alternatively, the inability to identify diagnostic features of an object might result in a slowed and unguided visual search when attempting to name an object. Originally, we hypothesized the first pattern of behaviors and predicted a more sporadic visual search approach. However, our results suggest that for words that were named incorrectly patients demonstrated the second pattern of behavior described, showing a slowed search strategy with fewer fixations in the given window.

The observed gaze patterns differ from our original predictions and from previous work in several respects. First, patients fixated less often for items they could not name, whereas related work using more complex visual arrays has demonstrated *more* fixations for forgotten items (Seckin et al., 2016). This discrepancy between studies could have resulted from different task demands. Consider the tasks involved in studies discussed: Rösler et al. (2000) visual-search task among letters and numbers, and Seckin et al. (2016) picture-word matching task. Both of these paradigms required patients to select an item from an array of competing stimuli. Reduced top-down semantic support for naming compelled patients to make a probabilistic selection based on bottom-up guided visual search. As a result, patient selections were characterized by numerous revisits among competing stimuli. In contrast, visual confrontation naming in our study involved only a single stimulus per trial with no extrinsic competition between picture stimuli on any individual trial. This methodological discrepancy between studies may account for the observed differences in the amount of fixation. In the current study, inaccurate naming (i.e., forgetting) was associated with fewer fixations. This pattern may reflect inability to effectively seek and focus on diagnostic semantic features necessary to accurately name a target picture. A similar interpretation of “feature dimming” was offered by Bozeat et al. (2003) in reference to altered picture drawing (e.g., drawing a duck with four legs) documented as their longitudinal patient cohort declined over time.

Another possible account of our finding that fewer fixations predicts when items are unknown relates to a division of labor in visual object recognition between global visual form (e.g., shape) vs. local visual detail (e.g., texture, facial features) (Bar, 2003; Bar et al., 2006). During picture viewing, simultaneous detail is available both from low and high spatial frequencies (Bar, 2003; Bar et al., 2006). Low spatial frequency details about global visual form (e.g., shape) can be assessed without fixating on the picture. In contrast, local visual form (e.g., texture) is represented by high spatial frequency detail, often requiring fixation(s) in numerous places. A neurotypical person names pictures both rapidly and accurately because they can effectively integrate low and high spatial frequency information. In contrast, the lack of top-down semantic support might compel the patient to conduct an unfocused search, attending to low spatial frequencies. Such a search strategy would be characterized by a high saccade count with a correspondingly low fixation count.

In a companion study we observed the longitudinal eye gaze patterns of objects that are consistently known, vulnerable to being forgotten, and objects that are consistently forgotten over the course of a 2-year study. The stimuli and procedure of presentation were identical to the current study. This companion analysis found a u-shaped pattern of eyetracking as objects go from known to vulnerable to forgotten. When the objects are known, the fixation count is low suggesting a streamlined and efficient top-down visual search. For words that are vulnerable to being forgotten the fixation count spikes and indicates that the patients are attempting to name the item by fixating on many different places to find the important features. However, once the words progress from being vulnerable to being completely forgotten, the fixation count drops below that of known words. This finding suggests that a low fixation count is a behavior that can result from two different stages of progressive anomia: a streamlined and organized visual search resulting in effective naming, or a slowed and unguided search resulting in incorrect naming (Reilly et al., under review). The eyetracking pattern for the latter stage of progressive anomia supports our finding that a low fixation count can in fact predict unknown words, although not words that are vulnerable and on the trajectory of being forgotten.

### Neuropsychological Correlations With Eyetracking

We assessed global cognition, working memory, language, semantic memory, and attention using a variety of offline neuropsychological measures. Patients showed correlations between online measures of eyetracking during naming with several of these offline neuropsychological measures (see Figure 2). Significant correlations were observed between saccade velocity MoCA score, digits backward, and Trail B time (in seconds). Significant correlations were also observed between saccade count and MoCA score. Additionally, a significant correlation was found between the MoCA and fixation count. However, this correlation did not follow a linear distribution and should be interpreted with caution.

Slowed saccade velocity predicted naming accuracy, and gaze slowing occurred in conjunction with declines in global cognition. Changes in saccade velocity could have either a cognitive or motor etiology. Lueck et al. (2000) found that patients with AD exhibit irregular saccades (e.g., more forward saccades per line and more saccadic regressions) during text reading compared to controls. Lueck et al. (2000), among other authors, have also found that increased saccadic abnormalities are correlated with a more severe cognitive impairment (Schewe et al., 1999). These studies link saccade behavior to difficulties with lexical-semantic access in AD. In contrast, a relative minority of studies have linked abnormal saccade behavior in AD to oculomotor dysfunction (Hutton et al., 1979; Pirozzolo and Hansch, 1981). Although oculomotor dysfunction is a plausible cause of saccade slowing, the observed correlations with declining global cognition suggest more of a cognitive etiology in our patient cohort (see also Scinto et al., 1994).

### Limitations

We did not observe expected correlations between neuropsychological tasks and eyetracking data, as we predicted a positive correlation between eyetracking patterns and the tests measuring semantic knowledge and naming ability. While the BNT showed some variation (1–14), this variation came from only two patients out of this cohort. Eight patients performed consistently at floor performance with little variance. This indicates that the patients included in this study began with an impaired semantic understanding. We did in fact see a correlation between eyetracking patterns and the MoCA. Since the MoCA is a measure of global cognition that assesses more than just the semantic impairment, these scores might not have started low but rather showed a progressive decline over time as patients became more impaired.

Secondly, although previous studies have ruled out oculomotor difficulties in their eyetracking studies (Scinto et al., 1994), we did not have our own experiment to rule out this possibility in our own cohort of patients.

In the current paper we dichotomized the data as either known (e.g., accurate) or unknown (e.g., inaccurate) and collapsed across time points in order to determine if there are eyetracking patterns that can predict accuracy. We recognize that is does not explicitly examine the change over time, although we believe it has important implications for such a longitudinal investigation. Reilly et al. (under review), described above, conducted this longitudinal analysis in the same cohort of patients.

Furthermore, we acknowledge that access-based anomic errors are common in PPA (Mesulam et al., 2009). While it is clear that the patients in our cohort exhibit progressive anomia, it is not as clear whether this is due to a semantic impairment or other causes (e.g., lexical access). In Table 1 we report scores for the neuropsychological task, Pyramid and Palm Trees (PPT), that assess semantic knowledge. Further examination of semantic vs. lexical access impairments would be useful in determining the cause of anomia in this cohort of patients and strengthen our findings.

### Clinical Implication

In all, these results support the notion that individuals with progressive anomia demonstrate specific gaze patterns for preserved concepts vs. impoverished concepts in naming tasks.

Though previous studies have characterized naming capabilities in PPA, no studies until now have identified eyetracking behaviors uniquely associated with known vs. forgotten items in progressive anomia. Such findings hold promise for the use of eyetracking as a clinical tool capable of identifying impoverished concept knowledge in progressive anomia. This finding has vast clinical implications for personalized language interventions. Recent work has advocated for the use of maintenance-based interventions over compensatory or restorative interventions, as *maintaining* a lexicon is more efficacious than *relearning* a lexicon for patients with progressive semantic degradation (Reilly, 2016). Using the approach of eyetracking during picture naming, therapists may be able to create patient-specific inventories of “at-risk” target words at the onset of treatment. With the ability to reliably predict which words will drop from a patient’s lexicon, interventions could adjust focus on an item-specific basis. Such treatments could maximize the prolongation of preserved concept knowledge and provide patients and their families with a personalized treatment that would help to maintain communication for as long as possible.

### Future Directions

Future work ought to specifically examine item-specific gaze patterns associated with items as they transition from known to unknown. There remains to be a comprehensive account of gaze patterns illustrating the progression of concept degradation, which inevitably leads to naming impairment. This work could lead to the use of gaze metrics as a cost-effective, mobile tool for preclinical identification of semantic impairment. Pairing this personalized treatment with a non-invasive brain stimulation that has been shown to increase naming speed and improve visual search (Binney et al., 2018), might further augment the benefit that this eyetracking treatment would exhibit on the naming performance of the patients. Binney et al. (2018) demonstrated that the use of transcortical direct current stimulation (tDCS) improves patients’ ability to locate salient features of an object during confrontation naming.

## Conclusion

This study demonstrates that eyetracking is a useful tool to detect the degradation of concept knowledge, as our results show that saccade velocity and the amount of fixations and saccades are significant predictors of unknown items. This information could be used to develop clinical therapies for progressive anomia; a devastating symptom with currently very few treatments.

## Data Availability Statement

The dataset for this study can be found in Open Science Framework (https://mfr.osf.io/render?url=https://osf.io/eutr2/?action=download%26mode=render).

## Ethics Statement

The studies involving human participants were reviewed and approved by Institutional Review Boards of Temple University and the University of Pennsylvania.

## Author Contributions

JR and DM contributed to the design of the study. MU collected the data. MU and MF organized the database. JR, DM, and MF performed the statistical analyses. BZ contributed to the statistical analyses. MU, MF, DM, and JR contributed to the writing and editing of the drafts.

## Conflict of Interest

The authors declare that the research was conducted in the absence of any commercial or financial relationships that could be construed as a potential conflict of interest.

## Footnotes

1 https://mfr.osf.io/render?url=https://osf.io/eutr2/?action=download%26mode=render
